# Evaluating the use of taxonomy in the IUCN Red List

**DOI:** 10.1111/cobi.70306

**Published:** 2026-05-21

**Authors:** Stephen T. Garnett, Claire E. Johnston, Phil Bowles, Les Christidis, Stijn Conix, J. W. Duckworth, Aaron Lien, Frank E. Zachos, Joeri Witteveen

**Affiliations:** ^1^ Research Institute for the Environment and Livelihoods Charles Darwin University Casuarina Northern Territory Australia; ^2^ IUCN Biodiversity Assessment Unit Gland Switzerland; ^3^ Southern Cross University Coffs Harbour New South Wales Australia; ^4^ Institut Supérieur de Philosophie Université Catholique de Louvain Louvain‐La‐Neuve Belgium; ^5^ IUCN SSC Asian Species Action Partnership Bristol UK; ^6^ School of Natural Resources and the Environment University of Arizona Tucson Arizona USA; ^7^ Natural History Museum Vienna Austria; ^8^ Department of Evolutionary Biology University of Vienna Vienna Austria; ^9^ Department of Genetics University of the Free State Bloemfontein South Africa; ^10^ Section for History and Philosophy of Science, Department of Science Education University of Copenhagen Copenhagen Denmark

**Keywords:** Catalogue of Life, CITES, governance, migratory species, species specialist groups, Catálogo de la Vida, CITES, especies migratorias, gobernanza, grupos de especialistas en especies, 治理, 物种专家组, 生命物种名录, 濒危野生动植物种国际贸易公约(CITES), 迁徙物种

## Abstract

Taxonomy defines the units that conservationists strive to preserve for future generations. However, the discovery of new species and the taxonomic revision of existing species affect conservation efforts. Despite the importance of taxonomy for a species’ conservation, there is currently no overview of how those leading species extinction risk assessments use taxonomy. To fill this gap, we asked the chairs of the International Union for Conservation of Nature's (IUCN) Species Survival Commission Specialist Groups and other IUCN expert groups that coordinate extinction risk assessments about the state of taxonomy within their taxonomic group of interest, their sources of information, and what taxonomy‐related challenges they encounter in conducting assessments. Most experts answered immediately and in detail, reflecting the importance of taxonomy. Approaches to taxonomy were diverse. Although most experts ignored debates about species definitions, for some groups, particularly mammals and birds, there were theoretical differences about what constitutes a species. Governance of species lists varied from individual experts making all decisions, to dedicated committees, to collective decisions for all taxonomic resolutions being made by others outside the group. The extent to which current taxonomy represents the views and consensus of the taxonomic community with an interest in that taxonomic group is not known. However, the taxonomies used in IUCN assessments differ from those used by other species conservation initiatives led by multilateral environmental agreements or the Catalogue of Life. Greater alignment is possible and could be beneficial, though it may constrain the flexibility and adaptability in taxonomic decision‐making that informs IUCN Red List assessments. We argue that regardless of the extent to which further alignment is sought, there is potential to enhance taxonomic list governance within the IUCN.

## Abstract (es)

Evaluación del uso de la taxonomía en la Lista Roja de la UICN

**Resumen:**

La taxonomía define las unidades que los conservacionistas se esfuerzan por preservar para las generaciones futuras. Sin embargo, el descubrimiento de nuevas especies y la revisión taxonómica de las especies existentes afectan a los esfuerzos de conservación. A pesar de la importancia de la taxonomía para la conservación de una especie, actualmente no existe una visión general de cómo utilizan la taxonomía quienes realizan las evaluaciones del riesgo de extinción de las especies. Para subsanar esta carencia, preguntamos a los presidentes de los Grupos de Especialistas de la Comisión de Supervivencia de Especies de la Unión Internacional para la Conservación de la Naturaleza (UICN) y a otros grupos de expertos de la UICN que coordinan las evaluaciones del riesgo de extinción sobre el estado de la taxonomía dentro de su grupo taxonómico de interés, sus fuentes de información y qué retos relacionados con la taxonomía encuentran al realizar las evaluaciones. La mayoría de los expertos respondieron de inmediato y con detalle, lo que refleja la importancia de la taxonomía. Los enfoques taxonómicos fueron diversos. Si bien la mayoría de los expertos ignoraron los debates sobre las definiciones de las especies, en el caso de algunos grupos, especialmente los mamíferos y las aves, existían diferencias teóricas sobre lo que constituye una especie. La gestión de las listas de especies variaba desde expertos individuales que tomaban todas las decisiones, pasando por comités especializados, hasta decisiones colectivas en las que todas las resoluciones taxonómicas eran adoptadas por personas ajenas al grupo. Se desconoce en qué medida la taxonomía actual representa las opiniones y el consenso de la comunidad taxonómica interesada en ese grupo taxonómico. Sin embargo, las taxonomías utilizadas en las evaluaciones de la UICN difieren de las empleadas por otras iniciativas de conservación de especies lideradas por acuerdos multilaterales sobre el medio ambiente o por el Catálogo de la Vida. Es posible lograr una mayor armonización, lo que podría resultar beneficioso, aunque podría limitar la flexibilidad y la adaptabilidad en la toma de decisiones taxonómicas que fundamentan las evaluaciones de la Lista Roja de la UICN. Sostenemos que, independientemente del grado de armonización que se busque, existe potencial para mejorar la gestión taxonómica dentro de la UICN.

## 评估《国际自然保护联盟(IUCN)濒危物种红色名录》中分类学的应用

**【摘要】** 分类学界定了保护工作者致力于为后代保存的保护单元。然而, 新物种的发现以及现有物种的分类修订都会影响保护工作。尽管分类学对物种保护至关重要, 但目前尚缺乏关于物种灭绝风险的主流评估如何运用分类学的总体概述。为填补这一空缺, 我们向国际自然保护联盟(IUCN)物种生存委员会各专家组主席, 以及其他负责协调灭绝风险评估的IUCN专家组成员, 征询了以下问题:在其关注的类群中, 分类学的现状如何;信息来源有哪些;以及在开展评估过程中遇到了哪些与分类学相关的挑战。大多数专家都迅速且详细地作出了回应, 这反映了分类学的重要性。结果表明, 各专家组对分类学的运用方法各不相同。虽然大多数专家忽略了关于物种定义的争论, 但对于某些类群(特别是哺乳动物和鸟类)而言, 关于物种的构成仍存在理论分歧。物种名录的管理方式也存在差异, 有的小组由单个专家全权决定, 有的小组由专门委员会负责, 还有的小组由外部的其他人员共同决定所有分类学问题。目前尚不清楚现行分类学在多大程度上代表了关注该类群的分类学界的观点和共识。然而, IUCN评估中采用的分类体系, 与其他由多边环境协定主导的物种保护倡议或生命物种名录数据库所采用的分类体系均存在差异。在分类学问题上进一步协调一致是可行且有潜在益处的, 尽管这可能会限制指导IUCN红色名录分类决策的灵活性和适应性。我们认为, 无论在多大程度上寻求进一步的协调一致, 都将有潜力改进IUCN内部的分类学管理方式。**【翻译:胡怡思;审校:聂永刚】**

## INTRODUCTION

The International Union for Conservation of Nature (IUCN) Red List of Threatened Species offers comprehensive data on the extinction risk of species on a global scale (Mace et al., 2018; Rodrigues et al., 2006). Over its 60‐year history, it has become a vital resource for research on biodiversity and a cornerstone for species conservation policy and action. What is more, the IUCN Red List has become a cornerstone in an ecosystem of other biodiversity knowledge products. For example, the IUCN Red List is the basis for the IUCN Red List Index (RLI) and the metric for species threat abatement and restoration (STAR), which are critical for tracking and mobilizing conservation efforts in the Kunming–Montreal Global Biodiversity Framework under the Convention on Biological Diversity (Butchart et al., 2025; CBD, 2022; Mair et al., 2021). It is also an important part of the EDGE approach to identify evolutionarily distinct and globally endangered species (Gumbs et al., 2023; Isaac et al., 2007).

The assessment of species for the IUCN Red List follows a highly structured process, outlined in a detailed protocol and accompanied by software tools, training courses, and workshops (IUCN, 2016). Notably, most assessments are conducted voluntarily by dedicated scientists worldwide. In principle, anyone familiar with red‐listing procedures can submit an assessment for review to the IUCN Red List Unit, which serves as the gatekeeper for the list. In practice, most assessments are carried out by the taxon‐specific specialist groups (SGs) of the IUCN's Species Survival Commission (SSC). Most of these SGs also serve as IUCN Red List authorities (RLAs)—that is, as the expert groups responsible for building assessment capacity and for ensuring that assessments within their scope adhere to the IUCN standards and guidelines. In addition to the over 100 SGs, the SSC also hosts several stand‐alone IUCN RLAs, such as the Brazilian Plant RLA, which assesses all Brazilian plant species and collaborates with BirdLife International as the RLA for all birds.

Before an extinction risk assessment for the IUCN Red List can be instigated, what qualifies as a species must first be determined. Although taxonomic status is undisputed for many species, definitions of what constitutes a species and how to delineate them can vary among taxonomists and evolve over time (Zachos, 2016). Taxonomic uncertainty, dissent, and change necessitate decisions about which taxonomic treatment to follow as a basis for IUCN Red List assessments (Mace, 2004).

Unlike the structured protocols and procedures for assessing species, the IUCN guidelines on taxonomic decision‐making are lean and flexible. Although requiring that taxa included on the IUCN Red List are “validly published in accordance with the appropriate international nomenclatural codes” (IUCN, 2016, p. 9), they allow for considerable bottom‐up discretion in choosing between competing taxonomic treatments. In exceptional cases, taxa that are not (yet) supported by a published source can be included on the IUCN Red List (IUCN, 2025). Within the list's governance structure, the responsibility for determining taxonomy lies with the RLAs. The current version of the Rules of Procedure for IUCN Red List Assessments (IUCN, 2016) notes that

The roles and responsibilities of the RLA include … serving as the taxonomic authority for species falling into the remit of the RLA … in other words, RLAs are responsible for determining and agreeing [on] the nomenclature used on the IUCN Red List for the species in that group, noting that RLAs, in turn, are subject to IUCN's own guiding principles on taxonomy (IUCN, 2016, p. 3).

These principles, which in 2016 were intended to become an annex to the IUCN Rules of Procedure once completed, had not been completed as of July 2025 (IUCN, 2025). In the meantime, some of the well‐resourced SGs developed their own checklists, and others handled taxonomic uncertainties or conflicts on a case‐by‐case basis. These choices can have far‐reaching consequences. We focused on how taxonomic decisions influence IUCN Red List risk categorizations and the extent to which the IUCN Red List aligns with other lists used by conservation policy makers, practitioners, and the broader public.

Taxonomic decisions can have a strong effect on how a species scores on a range of population‐level parameters, including population size, geographic range, and rate of decline, that codetermine its IUCN Red List status. Generally, the smaller the population of a species, the higher its risk of extinction. Therefore, when choosing between two equally valid taxonomic treatments for the species in a certain genus, opting for the treatment that recognizes a greater number of species is likely to result in higher risk categories being assigned to more species, which in turn has been argued to prevent the loss of biodiversity (Groves et al., 2010; May, 1990). In contrast, adopting a classification that consolidates those species into fewer, larger groups with wider distributions should be expected to lead to fewer species overall and to a higher proportion of those at lower risk. Although intuitive in theory, in practice, the few available case studies have yielded mixed results (Morrison III et al., 2009). There is a suggestion that elevated extinction risk assessments due to splitting have increased in mammals but the evidence is ambiguous; although Creighton et al. (2023) found no such results for primates, Amori and Luiselli (2024) did (as well as for Cetartiodactyla, but not for rodents). Results for birds were similarly equivocal (Simkins et al., 2020).

Taxonomic decisions that shape the IUCN Red List can also lead to misalignments with other checklists and taxonomic sources used by conservationists or other communities of practice. Apart from potentially causing confusion among and between user groups, such inconsistencies can also create regulatory loopholes or practical oversights that impede effective species conservation (Thomson et al., 2021).

Two processes affect taxonomic decision‐making in different ways. First, if a decision between different species delineations is guided by an anticipated assessment outcome, this could undermine scientific integrity (Conix et al., 2021) and lead to suboptimal resource allocation for overall extinction risk reduction. For example, splitting or merging groups merely with the intention of providing them with a favorable IUCN Red List status for conservation or commercial purposes should not be permitted. Units below the species level (defined taxonomically or geographically) can be included on the IUCN Red List under a different risk category than the species they are part of. However, the potential for disregarding something that is only a subspecies or only a subpopulation could provide a powerful incentive to interested parties to prefer species status. However, whether and to what extent anticipated outcomes of taxonomic choices can play a legitimate role remains an underexplored issue. Second, interoperability among taxonomies can improve efficiency (Feng et al., 2022). For example, if the Convention on International Trade in Endangered Species of Wild Flora and Fauna (CITES) and the IUCN Red List were to adopt the same lists, countries implementing IUCN and CITES guidelines would not have to reconcile different taxonomies in their legislation.

We aimed to enhance understanding of the complexities of taxonomic decision‐making, particularly in relation to its impacts on both effects of taxonomic decision‐making. First, to get a better understanding of taxonomic decision‐making within the IUCN, we surveyed most of the SGs to gain deeper insight into processes and parameters involved in taxonomic decision‐making across different SGs. Secondly, we mapped the similarities and differences between the taxonomic treatments adopted by the SGs (and their RLAs) and those used by other relevant lists in the same domain of taxonomy. Specifically, we compared the taxonomic reference sources of the SGs to those of two multilateral environmental agreements concerned with the conservation of species as well as to one comprehensive general list with broader user bases.

## METHODS

The websites of all SGs and IUCN RLAs within the IUCN SSC were assessed for information on taxonomy. This included the website itself and newsletters and other material attached to the site (Table S1). We obtained contact details of the chairs of all SGs and of the IUCN Red List authority coordinators (RLACs). For all groups concerned with the conservation of more than two species or where there was evidence of taxonomic uncertainty based on differences among lists, a semistructured interview was conducted by email. Each email conversation first asked what taxonomy a group was currently using “to understand which taxonomies the different SGs/RLAs use, where taxonomy is settled and where there are a variety of views.” The queries were individually tailored based on publicly available information and using existing knowledge about the principal source of taxonomic information used to define taxa for IUCN Red List assessment. Subsequent emails that failed to identify clearly the source of the taxonomic list underpinning the assessment for the group explored how taxonomic decisions were made. The questions asked were contingent on the answers from the first question. The aim was not to conduct replicable interviews; rather, it was to understand the situation at a particular juncture in time.

We compared the sources of lists used by the SGs with those used by CITES, the Convention on the Conservation of Migratory Species of Wild Animals (CMS), and the Catalogue of Life (COL). Both CITES and CMS are multilateral environmental agreements but have slightly different sources for their base taxonomy. CITES regulates trade in both plants and animals (but not yet fungi), so has an interest in taxonomy across a wider range of taxa than CMS, which is primarily concerned with transborder vertebrates, and currently includes only one invertebrate and no amphibians in its appendices. Parties to CITES sometimes agree to make exceptions to their baseline taxonomies for individual species, but we compared only the baseline taxonomies with those used by the IUCN. The COL has the broadest taxonomic scope, aiming to combine taxonomic lists for all the world's life forms and includes the lists of marine species aggregated by the World Register of Marine Species (WoRMS).

Summaries of the results were tabulated, and a report was circulated among all those interviewed. We then elicited further insights into taxonomic decision‐making in the IUCN that were also summarized where appropriate here.

## RESULTS

### Sample size and response rate

Of the 110 taxon‐specific SGs, six stand‐alone RLAs, and six national SGs contacted (Table S1), all but six responded. Often a response was received within minutes of sending the emails, with 43% received the day the query was sent. Many contained great detail, with much further information being received in follow‐up emails. From the speed and vigor of the responses, we infer that taxonomy was important to the IUCN SG community.

### Species concepts and taxonomic approaches

Only a small number of mammal SGs and the RLA for birds reported using a particular species concept as a basis for their taxonomic decision‐making. BirdLife International follows the biological species concept (BSC). The diagnostic phylogenetic species concept (dPSC) was adopted by two SGs as deliberate policy and by several others as default given the taxonomic sources they followed, although the number of these groups was uncertain. Two mammal SGs stated that they followed the BSC but that they aimed to conserve genetic diversity at the subspecies level. One SG described the confusion caused by adoption of the dPSC, which had led to uncertainty about the taxonomy used by the group. Although SGs responsible for other vertebrate groups had concerns about decisions concerning acceptance of species, none mentioned the species concept they adopted. No disputes about taxonomy were mentioned by SGs responsible for plants or invertebrates.

On a more pragmatic level, several groups adopted sets of (quasi‐)quantitative criteria for species delimitation. BirdLife International employed the Tobias et al. (2010) criteria, which combines morphological, behavioral, and acoustic differences to resolve uncertainties about species delimitation. Several mammal groups either adopted or are in the process of adopting a framework for species delimitation pioneered by the taxonomic task force of the Cat Specialist Group while revising the taxonomy of the Felidae (Kitchener et al., 2017). Informally known as the “traffic light system,” this framework scores taxonomic proposals on morphological, genetic, and biogeographic lines of evidence for recognizing taxonomic distinctiveness (Kitchener et al., 2022). Depending on the number and quality of independent lines of evidence available, a taxonomic proposal can be qualified as red (unlikely to be distinct), amber (taxon is contentious, further research required), or green (sufficient evidence for recognizing a distinct taxon). Other SGs that adopted this system have made different taxon‐relevant changes to the input criteria and weighting of those criteria.

### Reference taxonomies and decision‐making processes

**FIGURE 1:**
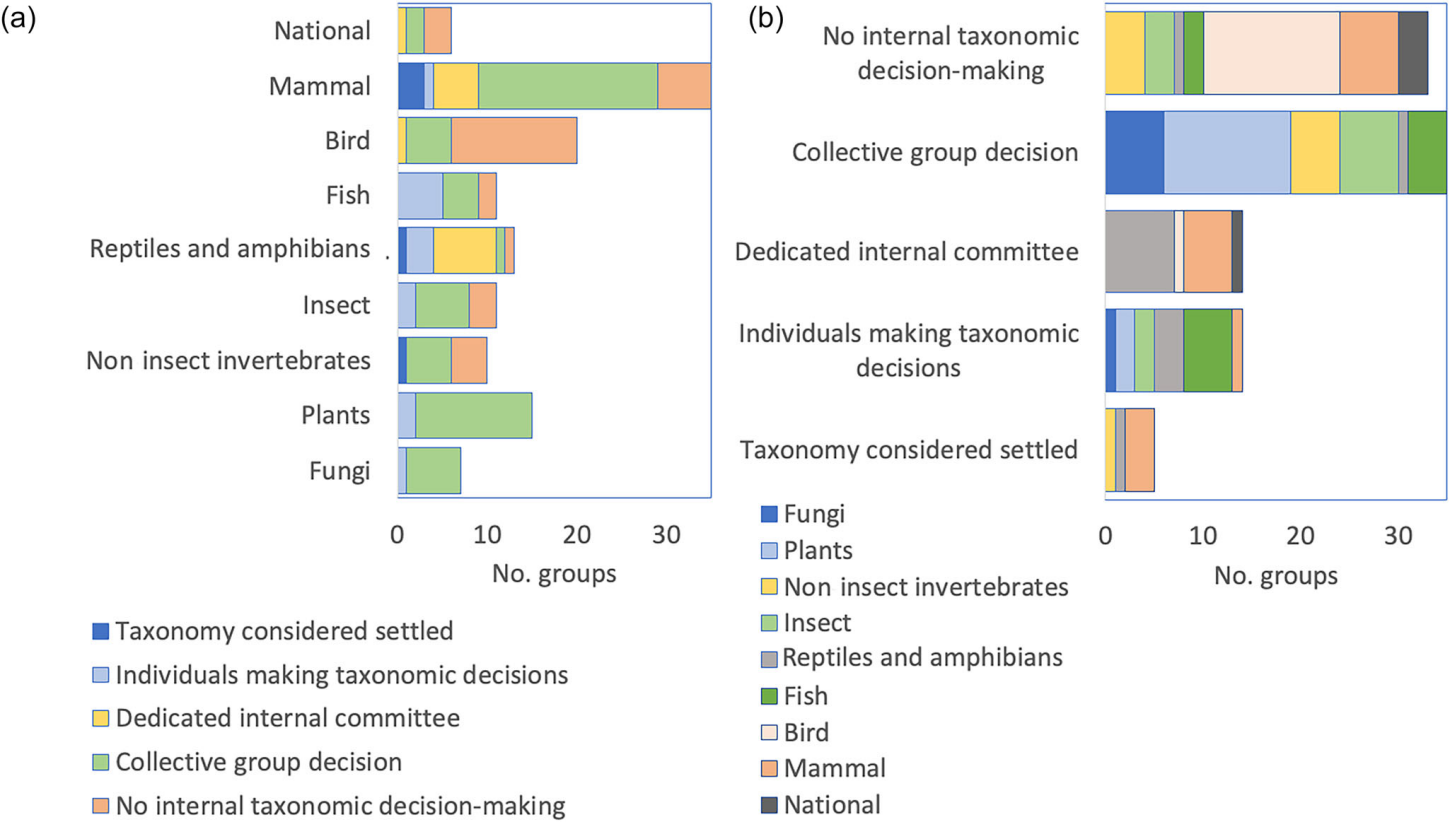
Sources of decisions about taxonomy used in International Union for Conservation of Nature (IUCN) Red List assessments by species specialist groups and other IUCN expert groups by (a) decision‐making process and (b) group type (‘national’ means ‘national species specialist groups’).

The ways in which decisions were made about taxonomy within each SG or RLA varied substantially (Figure 1). For five smaller groups (4%), the taxonomy was considered settled and there was no consideration of taxonomy. There were dedicated taxonomy committees in 14 SGs (11%), particularly those concerned with mammals and reptiles. A single individual made all taxonomic decisions in another 14 SGs (11%), including five of the 11 concerned with fishes. This was either at the behest of the group or because they were chair of the group and had taxonomic expertise. For about half the SGs (62, 51%), decisions appeared to be made by the group collectively, although individuals in some SGs appeared to be particularly influential in group decisions. The remaining 33 SGs (27%) drew their taxonomy from external sources uncritically.

For SGs relying on a single individual among their number, sometimes the chair, that person often had had a significant role in the group's taxonomy for some time. Comments included “I am also the primary taxonomy person for the group so most (if not all) descriptions come to me either formally or informally for review” and “I follow my taxonomy having spent my life studying and publishing about [the group].” Sometimes the taxonomy may not have been accepted by list aggregators but reflected the views of the expert based on their research: “I follow the taxonomy that I have published over the years (e.g., assessing the species that I have published as spp. novs. in the past). [the list aggregating body] mostly follows my taxonomy.” In some large and diverse SGs, there were several taxonomists: “Many members … are authorities on the taxonomy of their specific groups and so it is often better to leave the judgement to them.”

Taxonomic committees were particularly used by the SGs for mammals and reptiles. Many followed the aggregated lists but with minor deviations. Even if the same, some groups applied their own evaluative standards. For example, one mammal group described how, even though species listed by the group were currently the same as the Mammal Diversity Database (2025), they “decided against using that database without discussing the proposed taxonomic changes in our subcommittee because they may use other criteria than we do. For instance, our SG requires both molecular and morphological evidence for a taxonomic change to be accepted.”

Some SGs intended to establish committees but had not yet done so. For one SG, this was a matter of robustness to uncertainty: “future suggested taxonomic changes from elsewhere in the community … may be more controversial. As a result, I am hoping to get ahead of those issues by establishing a committee tasked with evaluating future change.”

The largest group of SGs were less specific about their taxonomic decision‐making but appeared to act collectively in deciding which taxonomy to follow but without specifying how the decisions were made. One group appeared to consist entirely of taxonomists “we are taxonomists, so we make our own taxonomic decisions and do not follow even [our institution] blindly.”

### Correspondence of SG lists to lists used by CITES, CMS, and COL

Because CITES and CMS cite the sources of their taxonomic information in their documentation, these could be compared with the sources used by the SGs for taxon groups that they had in common.

#### Convention on International Trade in Endangered Species of Wild Flora and Fauna

**FIGURE 2:**
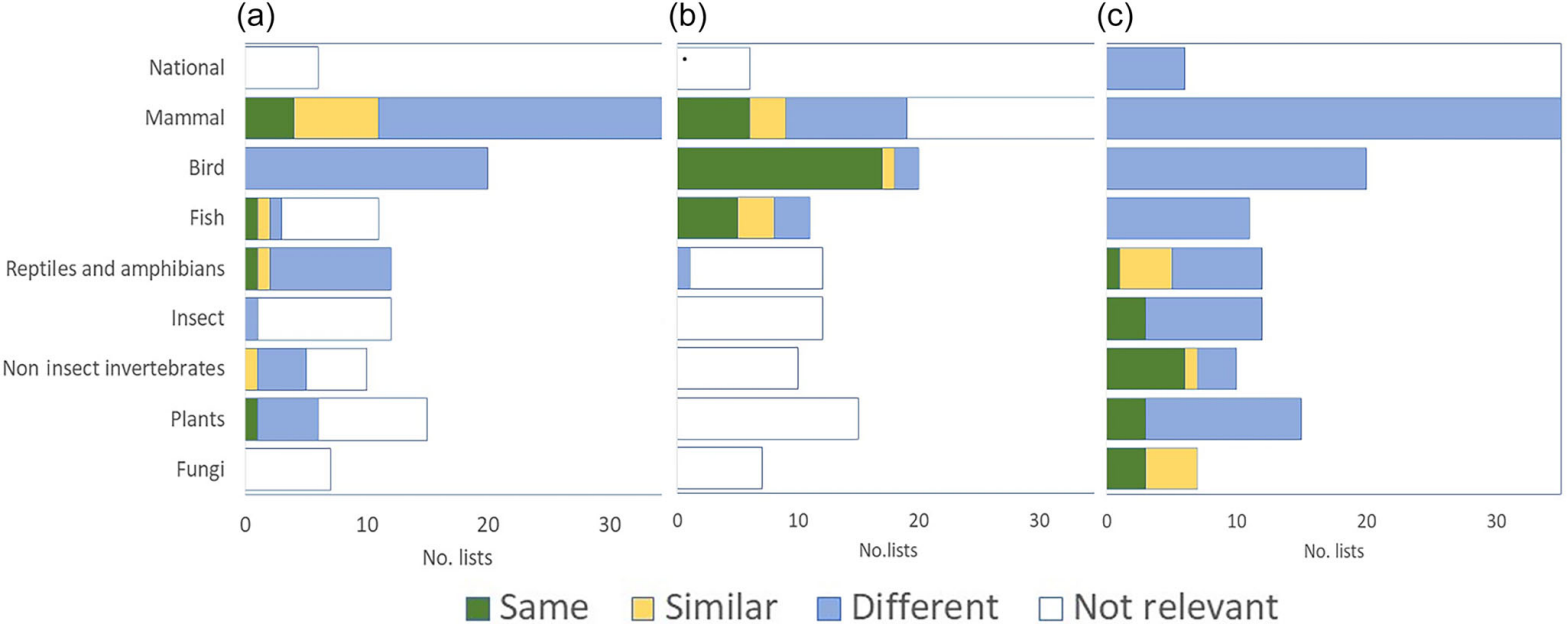
Differences between taxonomic sources used by International Union for Conservation of Nature (IUCN) Red List Species Specialist Groups and other IUCN expert groups and (a) the Convention on International Trade in Endangered Species of Wild Flora and Fauna, (b) Convention on Migratory Species, and (c) the Catalogue of Life ('national' means 'national species specialist groups'; 'not relevant' meansthat IUCN lists for taxa not covered by an international convention).

Of the 82 SGs concerned with CITES‐listed species, 68 (83%) did not use CITES taxonomic lists (Figure 2a). The low level of use of CITES taxonomy by the SGs for mammals was because few mammal groups used the principal source of taxonomic information cited by CITES (Wilson & Reeder, 2005). None of the bird SGs nor the IUCN Red List Authority for Birds (BirdLife International) used the CITES default sources of bird taxonomy (family level, Morony et al. [1975]; species and genera, Dickinson [2003, 2005]). Those maintaining the species taxonomy used by CITES chose not to join a collaboration among the three other major bird lists (BirdLife International [the IUCN Red List Authority for Birds], International Ornithological Community and eBird/Clements) to produce a single consolidated bird list: AviList (Rheindt et al., 2025). For fishes, only the Sturgeon SG explained explicitly that they adopted the CITES taxonomy (Catalog of Fishes) (Fricke et al., 2024), and the Shark SG used the Catalog of Fishes as their baseline. However, the Marine Fishes Red List Authority was of the view that fish taxonomy in general was now not covered adequately by either of the two major global lists of fish species (Catalog of Fishes, Fishbase). The Snake SG's snake taxonomy working group worked closely with the CITES nomenclature specialist to decide on controversial issues. The Amphibian Red List Authority used the Amphibians of the World (Frost, 2025) but made discretionary exceptions as it deems appropriate (as was done for some Australian species). There was no correspondence about the taxonomic source for the other groups; for plants and invertebrates, however, only the cycads had identical taxonomic sources for both lists despite increasing collaboration among plant taxonomists to produce consensus classifications (Klopper, 2025). The spider and scorpion taxonomy was similar, although the principal CITES taxonomic source was a decade old.

#### Convention on Migratory Species

Mammals, birds, fishes, marine turtles, and a single butterfly were listed by the Convention on Migratory Species (Figure 2b). Like CITES, CMS had Wilson and Reeder (2005) as its standard reference for mammals, which many SGs considered out of date. However, for marine mammals, CMS adopted the list published by the American Society for Marine Mammals, a list also favored by SGs focusing on marine mammal species. BirdLife International imposed its taxonomy on bird species listed under the IUCN Red List and was also the source of CMS taxonomy, resulting in a high level of correspondence, although a small number of SGs preferred other taxonomies for their own lists or used their own taxonomy even if it was identical to that of BirdLife. The adoption of AviList (Rheindt et al., 2025) would make this correspondence closer. Both CMS and many of the fish SGs continued to use the taxonomy of the Catalog of Fishes, though many had started to use alternative taxonomies in recent years in response to what they saw as a lack of editorial consensus at the Catalog. The difference for marine turtles arose because the Marine Turtle SG saw no need to cite a source for their taxonomy, whereas CMS, like CITES, did. The species listed were the same. The sole listed butterfly, the Monarch, was given no taxonomic source in either list of sources.

#### Catalogue of Life

The COL was in the process of assembling lists that, when complete, would include all species on Earth. Although not complete, the lists included over 2 million accepted species, and many more synonyms. However, historically it concentrated on obtaining lists for the invertebrate groups that make up the bulk of species across the globe and placed little emphasis on the lists of the species of higher public resonance that have tended to be of interest to those involved in SGs. Thus, mammals and birds were among 130 lists sourced from the Integrated Taxonomic Information System (ITIS), a global database of 868,000 scientific names, but neither of these lists reflected those used by any of the SGs for those groups (Figure 2c). Amphibians were drawn from the same database as that used by the IUCN Amphibian SG but that database was now several years out of date. Fishes came from Fishbase, a database that once used the Catalog of Fishes for its taxonomy but that did so no longer. There was a greater level of correspondence for the other groups, particularly for marine species, for which the COL drew from WoRMS, a taxonomist‐run aggregation of species lists that was the source of taxonomy for five of the seven SGs concerned primarily with marine species. Nevertheless, there were still many groups (103 groups, 84%) where the taxonomic source of the COL differed from the SGs.

## DISCUSSION

### Risk tolerance and evidential standards in taxonomic decision‐making

Although all SGs firmly grounded their taxonomic perspectives in biological data, their criteria, evidential standards, or thresholds for attributing species‐level distinctiveness varied. This variation arises partly from the adoption of different species concepts. In bird taxonomy, the problem was solved by adopting criteria that effectively impose the BSC on all the world's birds, which all bird SGs seem to accept even if some prefer to use an alternative baseline list. The issue is more vexed for mammals, where some SGs explicitly adopt a phylogenetic species concept, which historically has tended to result in the recognition of more species than does the BSC (Agapow et al., 2004), which is recognized by other groups. Although analyses of birds and primates suggest that splitting species is not necessarily associated with a rise in the number of threatened species and is not typically motivated by conservation concerns (Creighton et al., 2023; Simpkins et al., 2020; but see Amori & Luiselli [2024] for different results on the effects of splitting), the impression that different species concepts have been adopted for conservation purposes could damage public perceptions (Agapow et al., 2004).

However, the relevance of considering species concepts varies considerably among SGs. Across the different groups, explicit discussions of species concepts seem to be the exception rather than the rule. In addition, most species concepts can be operationalized in different ways, such that explicit endorsement of a species concept often says little about the resulting classification (Conix et al., 2023). Indeed, of the bird lists that have recently been integrated into one, the ones that adopt the BSC show substantial variation in the number of species they recognize, and no list recognizes more species than the one explicitly adhering to the PSC (Conix et al., 2024). Moreover, there need not always be much difference between the implications of species concepts for a final decision on what is recognized as a species. According to one SG chair, the phylogenetic species concept “…tends to increase the count, but phylogenetic studies can also lower the count.” Although explicit use of the phylogenetic species concept can result in the recognition of many species based on morphology, subsequent genetic analyses have then been used to differentiate those that warrant long‐term support from those that do not, so that the final taxonomy appears to represent a consensus among taxonomists that integrates different types of feature (e.g., van Elst et al., 2025). Similarly, with birds, morphological analyses that underpin the Tobias et al. (2021) approach have then been tested with genetic analyses, confirming many splits but negating others.

Regardless of which species concept, if any, is used, there is the need to decide on how much uncertainty to tolerate in recognizing groups as species on a red list (Stropp et al., 2022). Different attitudes toward the risk of error in making taxonomic determinations are clearly illustrated by the different implementations of the traffic light system and of integrative taxonomy. There are other SGs that express a preference for setting a comparatively lower bar for recognizing species‐level distinctiveness, for example, because time is of the essence. As one SG chair put it, “I'd be in favor of taking the just in case this novel proposal, which looks pretty sound, turns out to be valid, perhaps it makes sense to adopt it now, before corroboration and before it's bedded in generally because by the time the latter's happened it might be much more difficult to conserve it.” Another SG chair pointed out that SGs can be “nimble and unconstrained and can in effect adopt a new taxonomy at a moment's notice. They can be activist and can sometimes accept new taxonomic hypotheses that they subsequently have to reverse.” However, this high tolerance for commission errors is not shared by all SGs. Other SGs favor a less fluctuating, more robust taxonomy at the risk of more omission errors.

In addition to differing species concepts, a range of reasons and motives explain this variation in risk attitudes. For example, in some SGs, the size, motility, or accessibility to the (individuals from) taxa that fall within their remit pose considerable obstacles to collecting evidence at a level that other SGs can acquire with relative ease. Resource constraints also play a role. Although some SGs have the human and financial resources that allow them to fill gaps in the evidence record where needed, other SGs operate under severe constraints of taxonomic expertise and/or external funding.

Beyond the practical constraints on the amount of taxonomic evidence that can realistically be collected, some SGs also point to anticipated effects of taxonomy for conservation as a consideration in adopting a certain evidential standard. Some of the SGs that are willing to accept proposed splits of species based on a level of evidence that leaves room for discussion defend their choice by pointing to the risk of losing a species if its admission to the IUCN Red List as a separate taxon is postponed. Conversely, some of the SGs that intentionally set a much higher evidential bar likewise appeal to conservation impacts. They typically emphasize the need to minimize allocating scarce resources to species of questionable validity or point out that key conservation aims, such as increasing long‐term adaptive potential, are best supported by adopting stricter evidential standards. Finally, some SGs adopt a demanding approach to evidence primarily to mitigate the negative effects of taxonomic instability on conservation.

Close engagement with conservation impact is both relevant and often unavoidable, and responsible decision‐making demands that risk attitudes be shared and defended as much as possible. Moreover, lifting the veil on the complexity of taxonomic decision‐making under evidential uncertainty and the extent to which they are affected by values can help to identify the risk of *too* close engagement with conservation (Conix et al., 2021; Garnett & Christidis, 2007). If one were to adapt the IUCN Red List Conflict of Interest Policy's “practical examples of conflicts of interest that would lead to professional or financial gains from influencing any aspect of the Red List process” to taxonomy, they might read as follows: intentionally undersplitting a species because of concern that splitting would result in a higher category of threat, which could prompt a legislative response that negatively affects the operations or business of a company or jeopardizes the ability to undertake research on the species and intentionally oversplitting a species because the resulting higher category of threat improves an institution's ability to raise funds for research on, or conservation of, the species in question.

There was no evidence that such considerations influenced the taxonomy adopted by any SGs but, without an explicit statement explaining awareness of such potential conflicts of interest, and the measures put in place to avoid them, there could be an appearance of taxonomy being illicitly determined by conservation interests instead of taxonomy underpinning conservation.

### List governance

Some groups deferred all taxonomic decisions to a single individual whom they held in high regard. To what extent these individuals represented a consensus across whatever taxonomic community has an interest in the group was not clear. Some of these individuals were personally responsible for much of the modern taxonomy within the group of concern, so a taxonomic community might not exist. Many SG members spoke with respect and affection of individuals driving the creation of large databases even though they were gathering the information from large communities of contributing taxonomists, and even though they might disagree with those individuals on some minor points.

However, the working life and interest of individuals are limited, whereas committees have the potential to be durable and responsive to multiple members of a community. The taxonomic committees and task forces serving the SGs represent good examples of these. For example, the Australian Mammals Taxonomic Committee has a transparent, formal, and inclusive governance structure that is proving highly influential in mammal taxonomy generally as well as nationally, having been adopted by the Australian Government as its official source of taxonomic information for mammals. The global bird committee is in the process of adopting a governance structure that will allow its new consolidated global list to be perpetuated into the future.

### Taxonomic list engagement

There is a profound engagement between the SGs and the taxonomy of the groups with which they are concerned. The rapid and detailed responses to queries about taxonomy, and the detail provided, also show just how important taxonomy is to most SGs. Only a quarter of the SGs were content to defer to an external taxonomic list uncritically, rather than establishing a taxonomic position based on their own work or by drawing on several external published sources. Many of these were bird groups, but even there, where the final IUCN Red List follows the taxonomy of the Bird Red List Authority, BirdLife International, several groups prefer to follow their own taxonomic source to develop conservation strategies. Other groups that complied with an external list did so only because they thought they were obliged to and only learnt from this study that they had the freedom to do otherwise.

Considered from the viewpoint of the external dimension of list alignment, the SGs’ commitment to taxonomy is double‐edged. In some domains, it has led to the establishment of new checklists that have become the standard in their domain and removed the need for aligning the IUCN Red List with alternative lists. In other cases, the taxonomic freedom and agility of SGs have resulted in misalignments with external lists, either because they are not updated as frequently or because they are updated with a different evidential standard and risk attitude in mind (or both). In this context, it is worth bearing in mind that conventions like CITES, CMS, and, to some extent, COL tend to be slow and conservative. In CITES at least, there is a 3‐ to 10‐year review and deliberation process. The CITES list is intentionally conservative so that the revision process does not need to be repeated often because taxonomic change requires the approval of hundreds of parties to the convention (countries), any one of which may have political reasons for avoiding change.

There can be compelling financial and administrative reasons for some lists to be more risk averse about changing taxonomy, particularly where taxa are split. Many large databases, such as the BirdLife list or Fishbase, are fundamental to additional databases that describe distributions and other aspects of biology. Changing the taxonomy also requires that a host of other data are recalculated to comply with the new taxonomy, often in the face of sparse information. The same holds for conventions, such as CITES and CMS. For example, when CITES established its first list of threatened birds that might be affected by international trade, the Howard and Moore list (Dickinson, 2003) was thought by parties to the convention to have the most up‐to‐date taxonomy, well before BirdLife International produced its own list, which was then adopted by the IUCN. However, there has been a reluctance to change from the original source of taxonomy in CITES because of the web of legal consequences that are time‐consuming and expensive to change. The Convention on Migratory Species is a smaller convention than CITES, to which BirdLife provides valuable input, and was able to shift from an even older taxonomic source than Dickinson (2003) but only after three meetings of the convention parties held over 10 years. Even then, it succeeded only because of an intervention from the floor by a key delegate at the appropriate moment in committee when the proposal to shift taxonomies was on the brink of failure (S.T.G., personal observation).

The IUCN's *Guidelines for Using the IUCN Red List Categories and Criteria* could serve as a source of inspiration in this regard (IUCN Standards & Petitions Committee, 2024). The IUCN recognizes that each change in extinction risk category costs time and money and can potentially introduce errors that themselves take resources to correct. They therefore manage uncertainties around the many parameters used to assess extinction risk with a conceptually sophisticated, actionable approach to recognizing, representing, and dealing with uncertainties in data *about* species. We believe that there is an opportunity to take inspiration from these guidelines to provide advice on how to handle uncertainties in what constitutes a species for the purpose of red listing. That way, the often‐substantial social investment in the conservation of species that can be affected by taxonomic changes can be protected until changes fully satisfy the taxonomic community. The taxonomy itself may be sufficiently robust for it to be adopted by risk‐tolerant SGs, but those who need to implement changes to databases and the broader social structures built on those databases may appropriately be more risk averse.

### Prospects for alignment

Two processes are underway that will improve alignment. First, CMS adopted a process by which the taxonomy is reviewed against its standard list every 3 years. For birds, the CMS list has been that of BirdLife International, which reviews its list annually, a process that will continue now that BirdLife is a core partner of AviList (Rheindt et al., 2025). This means that the CMS bird list aligns perfectly with the IUCN and BirdLife list at the time the parties to the convention meet every 3 years, but not necessarily in between. Given that BirdLife has been somewhat conservative in its approach to taxonomy, the number of changes has been small and manageable. No party raised objections at the two conferences of the parties that have occurred since the process was introduced. There is an additional advantage in waiting 3 years before updating the CMS bird taxonomy as it allows for more consideration and refinement by the bird list compilers.

The second process is where there is a commitment to communication between the conventions, the IUCN SGs and the COL. Exact alignments between lists for groups like orthopterans or cycads are the result of proactive negotiation by the creators of those lists with those responsible for the larger lists, enhanced by close‐knit communities of taxonomists who have a common vision for their lists. The ornithologists, having had four competing lists a decade ago, now have two and those responsible for creating AviList have been negotiating to ensure it is adopted across multiple platforms and can be maintained into the future (Rheindt et al., 2025). The Mammal Diversity Database, which some SGs have adopted, is also providing a more comprehensive coverage of mammal species (Burgin et al., 2018, 2025). In a separate exercise, an assessment was undertaken of the quality of governance and contents of several lists that are used by the IUCN SGs (Garnett et al., 2026; Pape et al., 2026), and those most closely aligned across listing bodies tended to get high scores, though the sample is currently too small to yield statistically significant results.

There may also be cases where the benefits that might arise from complete alignment may not justify the cost. For example, CITES can decide whether to adopt taxonomic changes based not only on the latest literature but also on the practicalities of trade regulation. Debates on some taxonomic splits, such as between the two species‐level taxa of elephant *Loxodonta* occurring in Africa, have continued for many years and are still not fully agreed by parties to the treaty (CITES, 2025).

### Final remarks

The SGs of the IUCN SSC represent an extraordinary commitment from a huge number of individuals and institutions around the globe to minimizing extinction risk in the face of often ever‐increasing pressure. Taxonomy is fundamental to this effort. Many SG members are closely engaged in the taxonomy of their groups, often with preeminent knowledge. We have less information on whether the taxonomy espoused by SGs is consistent with that of other taxonomists, but there could be merit in groups assessing this themselves and articulating reasons where there are differences so that the reasons for differences are transparent. Some groups have committees making decisions, whereas some defer all taxonomic decisions to independent groups. Given the importance of taxonomy to the IUCN Red List, it is likely beneficial to ensure that the taxonomy used is of the highest possible quality and reflects the views of the relevant taxonomic community, while also considering the economic and conservation impacts that taxonomic changes may have on practitioners. There is also a need for clear and transparent processes in how taxonomic decisions are accepted or taken given the significant number of users of these lists.

This is also likely to lead to greater alignment between the major environmental conventions and the COL, which aggregates lists for multiple uses globally. This requires close collaboration among all parties, and the effort made by some SGs in achieving high levels of alignment, while also accommodating changes to taxonomy due to additional data, shows what can be achieved and pathways for doing so.

In addition to ensuring a sound evidential basis and an inclusive decision‐making process, there is value in reflecting on the risk attitudes and evidential standards that inform choices made in the face of uncertainty. The findings of this study suggest that strict prescriptions at the level of species concepts would be impractical and could risk alienating volunteer taxonomic communities that are already invested in their taxonomic approach. However, within a taxonomic group, a case could be made for aligning species concepts or taxonomic approaches. The ongoing adoption of the traffic light system by several mammal SGs points to the potential for developing a set of more flexible methodological and procedural recommendations that individual SGs could adapt to their taxon‐specific circumstances and available resources.

Overall, we consider that the IUCN taxonomy may well represent the consensus view of taxonomists for most groups but that, for some, there could beneficially be a provisional stage between a revision of taxonomy and its acceptance into the relevant databases, a stage during which there is review by whatever community of taxonomists exists for that group. Such reviews will commonly be performed by committees that have effective protocols for communicating their views among multiple groups that use taxonomic lists and for listening to the views of those groups.

## Supporting information



Supporting Information
