# Supplementary material for: Evaluating the use of taxonomy in the IUCN Red List
Source: Conserv Biol. 2026 May 21;40(5):e70306. doi: 10.1111/cobi.70306 (PMC13613567; doi:10.1111/cobi.70306)
Supplement: Supplementary file 1 [file COBI-40-e70306-s001.docx]

Supplementary Table 1. IUCN Species Specialist Groups and Red List Authorities with websites

| **Group** | **Group focus** | **Link** | **External Link** |
| --- | --- | --- | --- |
| Bird | Asian Song Bird Trade | https://www.iucn.org/our-union/commissions/group/iucn-ssc-asian-songbird-trade-specialist-group | https://www.asiansongbirdtradesg.com |
| Bird | Birds | https://www.iucn.org/our-union/commissions/group/iucn-ssc-bird-red-list-authority | https://www.birdlife.org |
| Bird | Bustard | https://www.iucn.org/our-union/commissions/group/iucn-ssc-bustard-specialist-group | |
| Bird | Cormorant | https://www.iucn.org/our-union/commissions/group/iucn-ssc-cormorant-specialist-group | http://cormorants.freehostia.com |
| Bird | Crane | https://www.iucn.org/our-union/commissions/group/iucn-ssc-crane-specialist-group | https://savingcranes.org/crane-specialist-group |
| Bird | Duck | https://www.iucn.org/our-union/commissions/group/iucn-ssc-duck-specialist-group | |
| Bird | Flamingo | https://www.iucn.org/our-union/commissions/group/iucn-ssc-flamingo-specialist-group | http://www.flamingo-sg.org |
| Bird | Galliformes | https://www.iucn.org/our-union/commissions/group/iucn-ssc-galliformes-specialist-group | |
| Bird | Goose | https://www.iucn.org/our-union/commissions/group/iucn-ssc-goose-specialist-group | https://www.geese.org/gsg/ |
| Bird | Heron | https://www.iucn.org/our-union/commissions/group/iucn-ssc-heron-specialist-group | https://www.heronconservation.org |
| Bird | Hornbill | https://www.iucn.org/our-union/commissions/group/iucn-ssc-hornbill-specialist-group | https://iucnhornbills.org |
| Bird | Loon | https://www.iucn.org/our-union/commissions/group/iucn-ssc-loon-specialist-group | |
| Bird | Pelican | https://www.iucn.org/our-union/commissions/group/iucn-ssc-pelican-specialist-group | https://www.wetlands.org/how-we-work/specialist-groups/pelican-specialist-group/ |
| Bird | Penguin | https://www.iucn.org/our-union/commissions/group/iucn-ssc-penguin-specialist-group | https://www.penguinsg.org/ |
| Bird | Pigeon and Dove | https://www.iucn.org/our-union/commissions/group/iucn-ssc-pigeon-and-dove-specialist-group | |
| Bird | Stork, Ibis and Spoonbill | https://www.iucn.org/our-union/commissions/group/iucn-ssc-stork-ibis-and-spoonbill-specialist-group | |
| Bird | Swan | https://www.iucn.org/our-union/commissions/group/iucn-ssc-swan-specialist-group | https://swansg.org/ |
| Bird | Threatened waterfowl | https://www.iucn.org/our-union/commissions/group/iucn-ssc-threatened-waterfowl-specialist-group | |
| Bird | Vulture | https://www.iucn.org/our-union/commissions/group/iucn-ssc-vulture-specialist-group | https://www.iucnvsg.org |
| Bird | Woodcock and Snipe | https://www.iucn.org/our-union/commissions/group/iucn-ssc-woodcock-and-snipe-specialist-group | https://www.wetlands.org/our-network/specialist-groups/woodcock-and-snipe-specialist-group/ |
| Fish | Anguillid Eel | https://www.iucn.org/our-union/commissions/group/iucn-ssc-anguillid-eel-specialist-group | https://www.zsl.org/what-we-do/projects/iucn-anguillid-eel-specialist-group-aesg |
| Fish | Freshwater Fish | https://www.iucn.org/our-union/commissions/group/iucn-ssc-freshwater-fish-specialist-group | https://freshwaterfish.org/ |
| Fish | Groupers and Wrasses | https://www.iucn.org/our-union/commissions/group/iucn-ssc-groupers-and-wrasses-specialist-group | |
| Fish | Salmonid | https://www.iucn.org/our-union/commissions/group/iucn-ssc-salmonid-specialist-group | |
| Fish | Seahorse, Pipefish and Seadragon | https://www.iucn.org/our-union/commissions/group/iucn-ssc-seahorse-pipefish-and-seadragon-specialist-group | Each species has a profile link to one or more of: Fishbase, GBIF, project seahorse, wikipedia, Encyclodeia of Life |
| Fish | Shark | https://www.iucn.org/our-union/commissions/group/iucn-ssc-shark-specialist-group | https://www.iucnssg.org/ |
| Fish | Snapper, Seabrim and Grunt | https://www.iucn.org/our-union/commissions/group/iucn-ssc-snapper-seabream-and-grunt-specialist-group | |
| Fish | Sturgeon | https://www.iucn.org/our-union/commissions/group/iucn-ssc-sturgeon-specialist-group | |
| Fish | Tuna and Billfish | https://www.iucn.org/our-union/commissions/group/iucn-ssc-tuna-and-billfish-specialist-group | |
| Fish | Croaker and Drum Fishes Red List Authority | IUCN SSC Croaker and Drum Fishes Red List Authority \| IUCN | |
| Fish | Marine Fishes Red list authority | IUCN SSC Marine Fishes Red List Authority \| IUCN | |
| Fungus | Brazil Fungal | https://www.iucn.org/our-union/commissions/group/iucn-ssc-brazil-fungal-specialist-group | |
| Fungus | Chytrid, Zygomycete, Downy Mildew and Myxomycete | https://www.iucn.org/our-union/commissions/group/iucn-ssc-chytrid-zygomycete-downy-mildew-and-myxomycete-specialist | http://www.cybertruffle.org.uk/moulds/index.htm |
| Fungus | Colombia Fungal | https://www.iucn.org/our-union/commissions/group/iucn-ssc-colombia-fungal-specialist-group | |
| Fungus | Cup-fungi, Truffles and Allies | https://www.iucn.org/our-union/commissions/group/iucn-ssc-cup-fungi-truffles-and-allies-specialist-group | |
| Fungus | Lichen | https://www.iucn.org/our-union/commissions/group/iucn-ssc-lichen-specialist-group | |
| Fungus | Mushroom, Bracket and Puffball | https://www.iucn.org/our-union/commissions/group/iucn-ssc-mushroom-bracket-and-puffball-specialist-group | |
| Fungus | Rusts and Smuts | https://www.iucn.org/our-union/commissions/group/iucn-ssc-rusts-and-smuts-specialist-group | http://www.cybertruffle.org.uk/rustsmut/ |
| Insect | Ant | https://www.iucn.org/our-union/commissions/group/iucn-ssc-ant-specialist-group | |
| Insect | Butterfly & Moth | https://www.iucn.org/our-union/commissions/group/iucn-ssc-butterfly-and-moth-specialist-group | |
| Insect | Cave Invertebrate | https://www.iucn.org/our-union/commissions/group/iucn-ssc-cave-invertebrate-specialist-group | |
| Insect | Dragonfly | https://www.iucn.org/our-union/commissions/group/iucn-ssc-dragonfly-specialist-group | https://worlddragonfly.org |
| Insect | Dung Beetle | https://www.iucn.org/our-union/commissions/group/iucn-ssc-dung-beetle-specialist-group | |
| Insect | Firefly | https://www.iucn.org/our-union/commissions/group/iucn-ssc-firefly-specialist-group | fireflyersinternational.net |
| Insect | Grasshopper | https://www.iucn.org/our-union/commissions/group/iucn-ssc-grasshopper-specialist-group | |
| Insect | Hoverfly | https://www.iucn.org/our-union/commissions/group/iucn-ssc-hoverfly-specialist-group | https://iucn-hsg.pmf.uns.ac.rs |
| Insect | Ladybird | https://www.iucn.org/our-union/commissions/group/iucn-ssc-ladybird-specialist-group | |
| Insect | Mayfly, Stonefly and Caddisfly | https://www.iucn.org/our-union/commissions/group/iucn-ssc-mayfly-stonefly-and-caddisfly-specialist-group | |
| Insect | Parasite | https://www.iucn.org/our-union/commissions/group/iucn-ssc-parasite-specialist-group | https://www.iucnparasites.com/our-mission |
| Insect | Wild Bee | https://www.iucn.org/our-union/commissions/group/iucn-ssc-wild-bee-specialist-group | |
| Mammal | African Elephant | https://www.iucn.org/our-union/commissions/group/iucn-ssc-african-elephant-specialist-group | |
| Mammal | African Rhino | https://www.iucn.org/our-union/commissions/group/iucn-ssc-african-rhino-specialist-group | |
| Mammal | Afrotheria | https://www.iucn.org/our-union/commissions/group/iucn-ssc-afrotheria-specialist-group | |
| Mammal | Anteater, Sloth and Armadillo | https://www.iucn.org/our-union/commissions/group/iucn-ssc-anteater-sloth-and-armadillo-specialist-group | |
| Mammal | Antelope | https://www.iucn.org/our-union/commissions/group/iucn-ssc-antelope-specialist-group | https://antelopesg.org |
| Mammal | Asian Elephant | https://www.iucn.org/our-union/commissions/group/iucn-ssc-asian-elephant-specialist-group | https://www.asesg.org |
| Mammal | Asian Rhino | https://www.iucn.org/our-union/commissions/group/iucn-ssc-asian-rhino-specialist-group | https://rhinos.org |
| Mammal | Asian Wild Cattle | https://www.iucn.org/our-union/commissions/group/iucn-ssc-asian-wild-cattle-specialist-group | https://www.asianwildcattle.org/species.html |
| Mammal | Australian Marsupial and Monotreme | https://www.iucn.org/our-union/commissions/group/iucn-ssc-australasian-marsupial-and-monotreme-specialist-group | |
| Mammal | Bat | https://www.iucn.org/our-union/commissions/group/iucn-ssc-bat-specialist-group | https://www.iucnbsg.org |
| Mammal | Bear | https://www.iucn.org/our-union/commissions/group/iucn-ssc-bear-specialist-group | https://globalbearconservation.org/Species |
| Mammal | Bison | https://www.iucn.org/our-union/commissions/group/iucn-ssc-bison-specialist-group | |
| Mammal | Canid | https://www.iucn.org/our-union/commissions/group/iucn-ssc-canid-specialist-group | https://www.canids.org |
| mammal | Caprinae | https://www.iucn.org/our-union/commissions/group/iucn-ssc-caprinae-specialist-group | iucncaprinaesg.weebly.com |
| Mammal | Cat | https://www.iucn.org/our-union/commissions/group/iucn-ssc-cat-specialist-group | www.catsg.org |
| Mammal | Cetacean | https://www.iucn.org/our-union/commissions/group/iucn-ssc-cetacean-specialist-group | https://iucn-csg.org/ |
| Mammal | Deer | https://www.iucn.org/our-union/commissions/group/iucn-ssc-deer-specialist-group | https://www.deerspecialistgroup.org/who-we-are/ |
| Mammal | Equid | https://www.iucn.org/our-union/commissions/group/iucn-ssc-equid-specialist-group | http://www.equids.org |
| Mammal | Giraffe and Okapi | https://www.iucn.org/our-union/commissions/group/iucn-ssc-giraffe-and-okapi-specialist-group | https://www.giraffidsg.org |
| Mammal | Hippo | https://www.iucn.org/our-union/commissions/group/iucn-ssc-hippo-specialist-group | https://www.hipposg.org |
| Mammal | Hyaena | https://www.iucn.org/our-union/commissions/group/iucn-ssc-hyaena-specialist-group | https://hyaenaspecialistgroup.org/2018/02/28/welcome-to-the-hyaena-specialist-group-website/ |
| Mammal | Lagomorph | https://www.iucn.org/our-union/commissions/group/iucn-ssc-lagomorph-specialist-group | https://www.lagomorphspecialistgroup.org |
| Mammal | New World Marsupial | https://www.iucn.org/our-union/commissions/group/iucn-ssc-new-world-marsupial-specialist-group | |
| Mammal | Otter | https://www.iucn.org/our-union/commissions/group/iucn-ssc-otter-specialist-group | https://www.otterspecialistgroup.org/osg-newsite/ |
| Mammal | Pangolin | https://www.iucn.org/our-union/commissions/group/iucn-ssc-pangolin-specialist-group | |
| Mammal | Peccary | https://www.iucn.org/our-union/commissions/group/iucn-ssc-peccary-specialist-group | https://www.iucn-wpsg.org/newsletter |
| Mammal | Pinniped | https://www.iucn.org/our-union/commissions/group/iucn-ssc-pinniped-specialist-group | |
| Mammal | Polar Bear | https://www.iucn.org/our-union/commissions/group/iucn-ssc-polar-bear-specialist-group | https://www.iucn-pbsg.org/ |
| Mammal | Primates | https://www.iucn.org/our-union/commissions/group/iucn-ssc-primate-specialist-group | |
| Mammal | Sirenia | https://www.iucn.org/our-union/commissions/group/iucn-ssc-sirenia-specialist-group | |
| Mammal | Small Carnivore | https://www.iucn.org/our-union/commissions/group/iucn-ssc-small-carnivore-specialist-group | https://smallcarnivore.org/ |
| Mammal | Small Mammal | https://www.iucn.org/our-union/commissions/group/iucn-ssc-small-mammal-specialist-group | https://small-mammals.org/ |
| Mammal | South American Camelid | https://www.iucn.org/our-union/commissions/group/iucn-ssc-south-american-camelid-specialist-group | https://www.camelid.org/index.php/es/ |
| Mammal | Tapir | https://www.iucn.org/our-union/commissions/group/iucn-ssc-tapir-specialist-group | https://tapirs.org/ |
| Mammal | Wild Pig | https://www.iucn.org/our-union/commissions/group/iucn-ssc-wild-pig-specialist-group | https://www.iucn-wpsg.org |
| Non-insect invertebrate | Coral | https://www.iucn.org/our-union/commissions/group/iucn-ssc-coral-specialist-group | https://www.coralspecialistgroup.org |
| Non-insect invertebrate | Freshwater Crustacean | https://www.iucn.org/our-union/commissions/group/iucn-ssc-freshwater-crustacean-specialist-group | |
| Non-insect invertebrates | Horseshoe Crab | https://www.iucn.org/our-union/commissions/group/iucn-ssc-horseshoe-crab-specialist-group | |
| Non-insect invertebrate | Marine Invertebrates Red list authority | IUCN SSC Marine Invertebrates Red List Authority \| IUCN | |
| Non-insect invertebrate | Marine Star | https://www.iucn.org/our-union/commissions/group/iucn-ssc-marine-star-specialist-group | |
| Non-insect invertebrate | Mite | https://www.iucn.org/our-union/commissions/group/iucn-ssc-mite-specialist-group | |
| Non-insect invertebrate | Mollusc | https://www.iucn.org/our-union/commissions/group/iucn-ssc-mollusc-specialist-group | |
| Non-insect invertebrate | Sea Cucumber | https://www.iucn.org/our-union/commissions/group/iucn-ssc-sea-cucumber-specialist-group | https://coastfish.spc.int/en/publications/bulletins/beche-de-mer/534-beche-de-mer-information-bulletin-42 |
| Non-insect invertebrate | Spider and Scorpion | https://www.iucn.org/our-union/commissions/group/iucn-ssc-spider-and-scorpion-specialist-group | |
| Non-insect invertebrate | Terrestrial and Freshwater Invertebrate Red List Authority | https://www.iucn.org/our-union/commissions/group/iucn-ssc-terrestrial-and-freshwater-invertebrate-red-list-authority | |
| Plant | Algae | https://www.iucn.org/our-union/commissions/group/iucn-ssc-seaweed-specialist-group | |
| Plant | Bryophyte | https://www.iucn.org/our-union/commissions/group/iucn-ssc-bryophyte-specialist-group | |
| Plant | Cactus & Succulent Plants | https://www.iucn.org/our-union/commissions/group/iucn-ssc-cactus-and-succulent-plants-specialist-group | |
| Plant | Carnivorous Plant | https://www.iucn.org/our-union/commissions/group/iucn-ssc-carnivorous-plant-specialist-group | www.iucn-cpsg.org |
| Plant | China Plants | https://www.iucn.org/our-union/commissions/group/iucn-ssc-china-plant-specialist-group | |
| Plant | Colombian Plant | https://www.iucn.org/our-union/commissions/group/iucn-ssc-colombian-plant-specialist-group | |
| Plant | Conifers | https://www.iucn.org/our-union/commissions/group/iucn-ssc-conifer-specialist-group | https://threatenedconifers.rbge.org.uk |
| Plant | Cuban Plants | https://www.iucn.org/our-union/commissions/group/iucn-ssc-cuban-plant-specialist-group | |
| Plant | Cycads | https://www.iucn.org/our-union/commissions/group/iucn-ssc-cycad-specialist-group | www.cycadgroup.org |
| Plant | Freshwater Plants | https://www.iucn.org/our-union/commissions/group/iucn-ssc-freshwater-plant-specialist-group | |
| Plant | Galapagos Plants | https://www.iucn.org/our-union/commissions/group/iucn-ssc-galapagos-plant-specialist-group | |
| Plant | Mangrove | https://www.iucn.org/our-union/commissions/group/iucn-ssc-mangrove-specialist-group | https://www.zsl.org/what-we-do/projects/mangroves |
| Plant | Orchid | https://www.iucn.org/our-union/commissions/group/iucn-ssc-orchid-specialist-group | https://www.orchidspecialistgroup.com |
| Plant | Palm | https://www.iucn.org/our-union/commissions/group/iucn-ssc-palm-specialist-group | |
| Plant | Seagrass | https://www.iucn.org/our-union/commissions/group/iucn-ssc-seagrass-species-specialist-group | |
| Reptile and amphibian | Amphibian | https://www.iucn.org/our-union/commissions/group/iucn-ssc-amphibian-specialist-group | |
| Reptile and amphibian | Chameleon | https://www.iucn.org/our-union/commissions/group/iucn-ssc-chameleon-specialist-group | |
| Reptile and amphibian | Crocodile | https://www.iucn.org/our-union/commissions/group/iucn-ssc-crocodile-specialist-group | http://www.iucncsg.org |
| Reptile and amphibian | Iguana | https://www.iucn.org/our-union/commissions/group/iucn-ssc-iguana-specialist-group | https://www.iucn-isg.org |
| Reptile and amphibian | Marine Turtle | https://iucn.org/sites/default/files/2022-10/2021-iucn-ssc-marine-turtle-sg-report_publication.pdf | https://www.iucn-mtsg.org |
| Reptile and amphibian | Monitor Lizards | https://www.iucn.org/our-union/commissions/group/iucn-ssc-monitor-lizard-specialist-group | https://iucn-mlsg.org |
| Reptile and amphibian | Sea Snake | https://www.iucn.org/our-union/commissions/group/iucn-ssc-sea-snake-specialist-group | |
| Reptile and amphibian | Skink | https://www.iucn.org/our-union/commissions/group/iucn-ssc-skink-specialist-group | : https://www.skinks.org |
| Reptile and amphibian | Snake | https://www.iucn.org/our-union/commissions/group/iucn-ssc-snake-specialist-group | https://www.iucnsnake.org/ |
| Reptile and amphibian | Snake and Lizard Red List Authority | https://www.iucn.org/our-union/commissions/group/iucn-ssc-snake-and-lizard-red-list-authority | |
| Reptile and amphibian | Tortoise and Freshwater Turtle | https://www.iucn.org/our-union/commissions/group/iucn-ssc-tortoise-and-freshwater-turtle-specialist-group | https://iucn-tftsg.org/taxonomic-literature-database/ |
| Reptile and amphibian | Viper | https://www.iucn.org/our-union/commissions/group/iucn-ssc-viper-specialist-group | https://viperconservation.org |
| Country | China Species | https://www.iucn.org/our-union/commissions/group/iucn-ssc-china-species-specialist-group | |
| Country | Colombia Species | https://www.iucn.org/our-union/commissions/group/iucn-ssc-colombia-species-specialist-group | |
| Country | New Caledonia Plant Red List Authority | IUCN SSC New Caledonia Plant Red List Authority \| IUCN | |
| Country | North American Plant Red List Authority | IUCN SSC North American Plant Red List Authority \| IUCN | |
| Country | Turkey Plant Red List Authority | IUCN SSC Turkey Plant Red List Authority \| IUCN | |
| Country | West Africa Plant Red List Authority | IUCN SSC West Africa Plant Red List Authority \| IUCN | |
